# Autoimmune hypothyroidism and endometriosis: evidence from bidirectional Mendelian randomization and an independent clinical cohort analysis

**DOI:** 10.3389/fendo.2026.1898299

**Published:** 2026-08-04

**Authors:** Jiawei Shi, Weijie Jiang, Hui Chen, Liming Zhou, Juwei Hu

**Affiliations:** 1 Department of Gynecology, Ningbo Women and Children's Hospital, Ningbo University, Ningbo, China; 2 Reproductive Medicine Center, Ningbo Women and Children's Hospital, Ningbo University, Ningbo, China

**Keywords:** autoimmune hypothyroidism, bidirectional analysis, clinical cohort analysis, endometriosis, genome-wide association study, Mendelian randomization, thyroid autoimmunity

## Abstract

**Background:**

Observational studies have reported comorbidity between autoimmune thyroid disease and endometriosis, but whether this reflects a direct causal relationship or shared susceptibility remains unclear. We evaluated the bidirectional relationship between autoimmune hypothyroidism (AIHT) and endometriosis using Mendelian randomization (MR) and an independent clinical cohort.

**Methods:**

Genome-wide association study (GWAS) summary statistics were obtained for AIHT from a FinnGen R12–UK Biobank meta-analysis and for endometriosis from GWAS Catalog study GCST90205183. Genome-wide significant instruments underwent linkage disequilibrium clumping, genome-build liftover, and harmonization. Inverse-variance weighted (IVW) analysis was primary, with weighted median and MR-Egger analyses as complements. Sensitivity analyses included heterogeneity and MR-Egger intercept tests, Mendelian randomization pleiotropy residual sum and outlier (MR-PRESSO), leave-one-out analysis, phenome-wide association study-informed filtering, and exclusion of the human leukocyte antigen region. Clinical associations were assessed using multivariable logistic regression.

**Results:**

After harmonization, 248 single-nucleotide polymorphisms (SNPs) were included for AIHT-to-endometriosis analysis and 26 for endometriosis-to-AIHT analysis. IVW showed no association of genetic liability to AIHT with endometriosis (odds ratio [OR] = 0.985, 95% confidence interval [CI]: 0.957–1.014, P = 0.315) or of genetic liability to endometriosis with AIHT (OR = 0.999, 95% CI: 0.951–1.049, P = 0.968). MR-PRESSO global tests were significant in both directions, indicating pleiotropic heterogeneity. No forward outlier was identified; removal of the reverse-direction outlier rs2433340 did not materially alter the estimate (corrected OR = 1.007, 95% CI: 0.960–1.056, P = 0.769). The cohort included 7,825 women: 853 with confirmed endometriosis and 6,972 diagnosis-negative controls. Endometriosis was not associated with AIHT after adjustment (OR = 0.753, 95% CI: 0.440–1.287, P = 0.299); findings were also non-significant for thyroid autoimmunity, thyroid peroxidase antibody positivity, thyroglobulin antibody positivity, and hypothyroidism.

**Conclusion:**

This integrated genetic and clinical analysis found no robust evidence that genetic liability to AIHT materially affects overall endometriosis risk or vice versa in the datasets analyzed, and no clear clinical association between confirmed endometriosis and AIHT or thyroid autoimmunity. Small effects and sex- or subtype-specific associations cannot be excluded.

## Introduction

Endometriosis is a chronic, estrogen-dependent gynecological disorder characterized by the presence of endometrium-like tissue outside the uterine cavity. It affects a substantial proportion of women of reproductive age and is associated with dysmenorrhea, chronic pelvic pain, infertility, impaired sexual function, and reduced quality of life (1, 2). Although retrograde menstruation, hormonal dependence, and ectopic lesion implantation remain central to its pathophysiological framework, endometriosis is increasingly recognized as a complex disorder with immune-inflammatory, hormonal, genetic, and systemic features. Recent large-scale genome-wide association studies (GWAS) have refined its genetic architecture and revealed shared genetic components with pain-related and inflammatory conditions, supporting the view that endometriosis extends beyond a purely localized pelvic disease (1).

Autoimmune hypothyroidism (AIHT), commonly related to Hashimoto thyroiditis, is one of the most prevalent organ-specific autoimmune diseases in women. It is characterized by thyroid autoimmunity, progressive thyroid functional impairment, and, in clinically manifest cases, biochemical hypothyroidism. Compared with circulating thyroid function traits or broad hypothyroidism phenotypes, AIHT more specifically captures thyroid dysfunction occurring in an autoimmune context. A recent GWAS meta-analysis of FinnGen and UK Biobank data identified multiple independent genetic signals for AIHT and showed that AIHT liability reflects both shared autoimmune susceptibility and thyroid-specific biological contributions (3). These advances provide a more disease-specific genetic proxy for autoimmune thyroid dysfunction.

A relationship between AIHT and endometriosis is biologically plausible. Endometriosis involves immune-inflammatory activation, altered innate and adaptive immune responses, and impaired clearance of ectopic endometrial tissue, whereas AIHT reflects loss of immune tolerance to thyroid antigens. Epidemiological and genetic studies have reported overlap between endometriosis and immune-mediated conditions (4). However, such overlap does not necessarily imply a direct causal relationship. Observed associations may arise from shared immune-inflammatory susceptibility, residual confounding, surveillance bias, reproductive health-seeking behavior, or reverse causation.

Previous Mendelian randomization (MR) studies of thyroid-related reproductive outcomes have mainly focused on thyroid function traits, such as thyrotropin and free thyroxine, or on broad thyroid disease phenotypes (5, 6). These exposures may capture hormonal regulation or heterogeneous thyroid dysfunction rather than autoimmune thyroid disease itself. Consequently, it remains unclear whether genetic liability to AIHT influences endometriosis risk, or whether genetic liability to endometriosis contributes to AIHT. A bidirectional two-sample MR design can help clarify these possible causal directions while reducing confounding and reverse causation (7). Because immune-mediated traits often involve shared genetic architecture and pleiotropic loci, sensitivity analyses addressing pleiotropy and immune-related genomic regions are essential for robust interpretation.

In the present study, we performed a bidirectional two-sample MR analysis using large-scale GWAS summary statistics to evaluate the potential causal relationship between AIHT and endometriosis. We complemented the genetic analysis with an independent retrospective infertility-clinic cohort to assess whether women with endometriosis had increased AIHT or thyroid autoimmunity in a real-world reproductive medicine setting. This clinical component was intended to contextualize the MR findings rather than to establish causality. This integrated design aimed to assess whether the reported epidemiological overlap between AIHT and endometriosis is consistent with a major direct causal relationship while considering shared immune-inflammatory background, residual confounding, and non-causal comorbidity as alternative explanations.

## Materials and methods

### Study design

We conducted an integrated genetic epidemiology and independent clinical cohort analysis to evaluate the relationship between AIHT and endometriosis. The study consisted of two complementary components. First, a bidirectional two-sample MR analysis was performed using publicly available GWAS summary statistics. In the forward analysis, genetic liability to AIHT was evaluated in relation to endometriosis risk. In the reverse analysis, genetic liability to endometriosis was evaluated in relation to AIHT risk. Second, an independent retrospective infertility-clinic cohort was analyzed to examine whether women with endometriosis had a higher prevalence of AIHT or thyroid autoimmunity in a real-world reproductive medicine setting.

**Figure 1:**
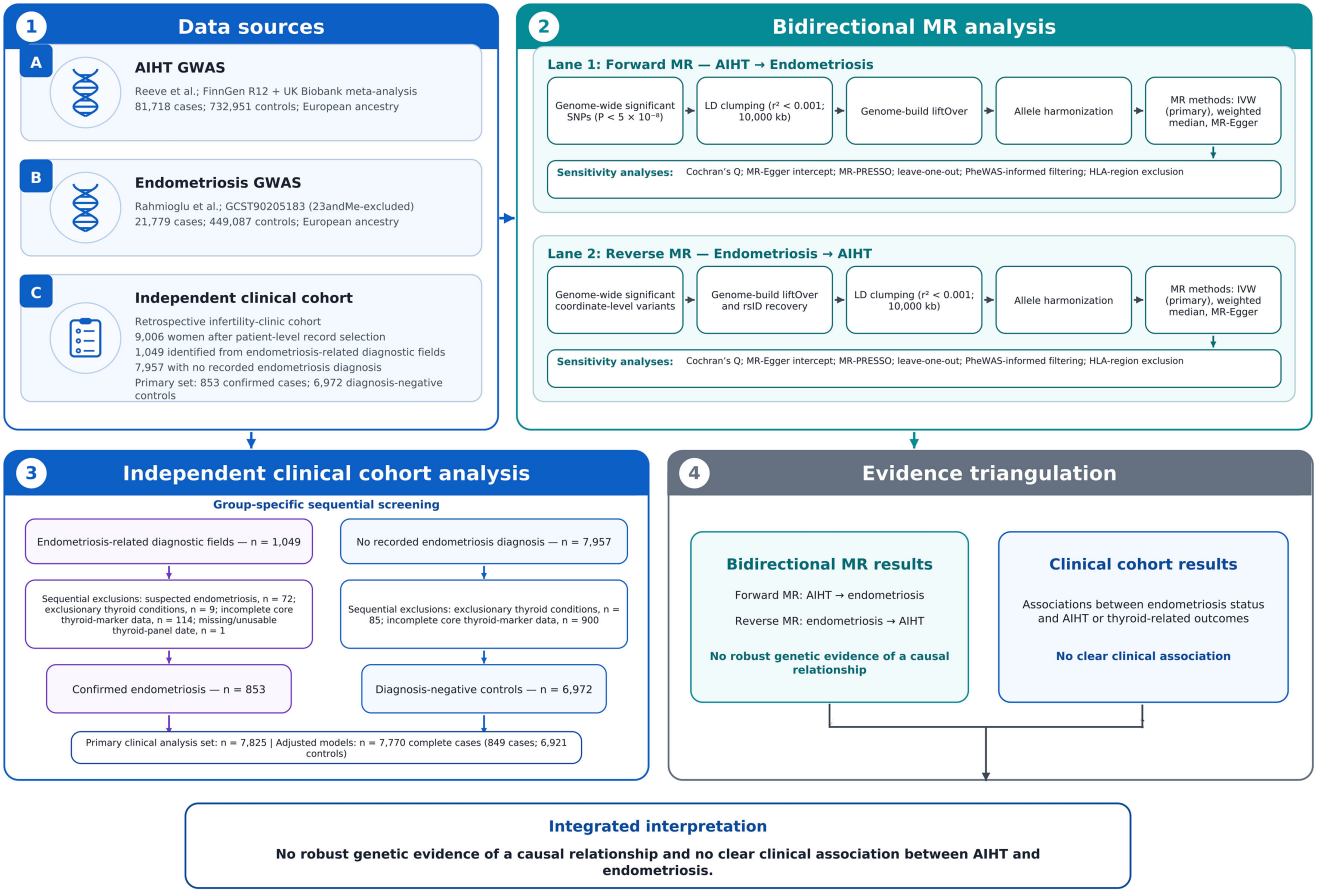
Study workflow of the integrated bidirectional Mendelian randomization and independent clinical cohort analysis. The workflow summarizes the integrated design used to investigate the relationship between autoimmune hypothyroidism (AIHT) and endometriosis. The study included a bidirectional two-sample Mendelian randomization (MR) analysis and an independent retrospective infertility-clinic cohort analysis. In the forward MR analysis, genetic liability to AIHT was evaluated in relation to endometriosis risk; in the reverse analysis, genetic liability to endometriosis was evaluated in relation to AIHT risk. Instrumental variables were selected using genome-wide significance thresholds and linkage disequilibrium clumping, followed by genome-build liftover, outcome matching, and allele harmonization. The independent clinical cohort was used to compare AIHT and thyroid autoimmunity between women with confirmed endometriosis and diagnosis-negative controls in a reproductive medicine setting.

The MR component was designed to evaluate genetic evidence for causality, whereas the clinical cohort analysis was intended to contextualize the MR findings by assessing clinical comorbidity patterns rather than to establish causality. The overall workflow is shown in **Figure 1**, and the data sources are summarized in **Table 1**. The MR component was reported with reference to the Strengthening the Reporting of Observational Studies in Epidemiology using Mendelian Randomization (STROBE-MR) guidelines.

**Table 1:** Data sources for Mendelian randomization and independent clinical cohort analysis.

Component	Data source	Role in the study	Sample size	Population
Autoimmune hypothyroidism GWAS	Reeve et al.; FinnGen R12 and UK Biobank meta-analysis	Exposure in forward MR; outcome in reverse MR	81,718 cases and 732,951 controls	European ancestry
Endometriosis GWAS	Rahmioglu et al.; GWAS Catalog GCST90205183	Outcome in forward MR; exposure in reverse MR	21,779 cases and 449,087 controls	European ancestry
Independent clinical cohort	Retrospective infertility-clinic cohort from the present study	Independent clinical cohort analysis	9,006 women, including 1,049 identified from endometriosis-related diagnostic fields and 7,957 with no recorded endometriosis diagnosis	Chinese infertility-clinic population
Primary clinical analysis set	Patient-level clinical cohort dataset	Main clinical cohort analysis	7,825 women, including 853 with confirmed endometriosis and 6,972 diagnosis-negative controls	Chinese infertility-clinic population

FT4, free thyroxine; GWAS, genome-wide association study; MR, Mendelian randomization; TgAb, thyroglobulin antibody; TPOAb, thyroid peroxidase antibody; TSH, thyroid-stimulating hormone.

Endometriosis status in the independent clinical cohort was identified from diagnostic fields and confirmed by laparoscopic biopsy according to institutional diagnostic records. The primary clinical analysis set included women with complete core thyroid markers and excluded suspected endometriosis, Graves’ disease, hyperthyroidism, thyroid cancer, thyroidectomy history and radioiodine treatment history. Core thyroid markers included TSH, FT4, TPOAb and TgAb.

### GWAS data sources

GWAS summary statistics for AIHT were obtained from the FinnGen R12–UK Biobank GWAS meta-analysis reported by Reeve et al. (3). The strict AIHT phenotype, E4_HYTHY_AI_STRICT, included 81,718 cases and 732,951 controls and was provided in Genome Reference Consortium Human Build 38 (GRCh38) coordinates according to FinnGen documentation (8).

GWAS summary statistics for endometriosis were obtained from the GWAS Catalog study accession GCST90205183, reported by Rahmioglu et al. (1). The publicly available 23andMe-excluded dataset included 21,779 European ancestry cases and 449,087 European ancestry controls and was provided in Genome Reference Consortium Human Build 37 (GRCh37) coordinates. These datasets were used reciprocally as exposure and outcome sources in the bidirectional MR analyses.

The 23andMe-excluded endometriosis dataset was used because full summary statistics including 23andMe participants were not publicly available. Participant-level identifiers and cohort-specific overlap counts were unavailable; therefore, the actual participant-overlap fraction between the two GWAS datasets could not be directly estimated. A scenario-based assessment of potential overlap-related bias was subsequently performed.

The AIHT summary statistics were derived from a sex-combined meta-analysis rather than a female-specific GWAS. In the original AIHT study, the FinnGen GWAS models adjusted for age, sex, genotyping batch and ancestry principal components, whereas the UK Biobank analyses included age, sex, age-by-sex, age-squared, age-squared-by-sex and ancestry principal components. These adjustments account for sex as a model covariate but do not provide female-specific single-nucleotide polymorphism (SNP)–AIHT association estimates. Accordingly, in the reverse MR analysis, SNP–endometriosis associations were derived from a female-specific phenotype, whereas SNP–AIHT outcome associations reflected a sex-combined population.

### Instrument selection and harmonization

Genome-wide significant variants associated with each exposure were selected using a threshold of P < 5 × 10^-8^. To obtain independent instruments, linkage disequilibrium (LD) clumping was performed using r^2^ < 0.001 within a 10,000-kb window based on a European ancestry LD reference panel. This stringent specification matches the clumping parameters used in the MR-Base framework and has also been adopted in published two-sample MR studies (9–11). It was selected to prioritize instrument independence and reduce the retention of correlated variants from broad association loci.

For the forward analysis, AIHT-associated variants were selected using reference SNP identifiers (rsIDs) from the AIHT GWAS, clumped for LD, and converted from GRCh38 to GRCh37 before matching to the endometriosis outcome dataset. Of the 15 variants that failed liftOver in the raw pre-clumping candidate pool, none was retained after LD clumping; consequently, these mapping failures did not affect the 274 independent AIHT variants taken forward for outcome matching. For the reverse analysis, 863 genome-wide significant endometriosis-associated coordinate-level variants were identified from the released endometriosis summary statistics using chromosome, position, and allele information because rsIDs were not directly available. All 863 variants were successfully converted from GRCh37 to GRCh38. The lifted coordinates were then matched to the AIHT GWAS reference, and non-empty rsIDs were recovered for all 863 variants. These variants constituted the pre-clumping instrument set. After LD clumping, 27 independent endometriosis instruments were retained; 26 remained after allele harmonization and were included in the reverse MR analysis.

Genome-build conversion was performed using University of California, Santa Cruz liftOver chain files through the rtracklayer package (12, 13). Exposure and outcome datasets were harmonized using TwoSampleMR to ensure that effect estimates corresponded to the same effect allele. Palindromic SNPs with intermediate allele frequencies were removed according to the default TwoSampleMR harmonization rules to minimize strand ambiguity. After harmonization, 248 SNPs were retained for the AIHT-to-endometriosis analysis and 26 SNPs for the endometriosis-to-AIHT analysis. The instrument selection and harmonization process is summarized in **Table 2**, and harmonized SNP-level data are provided in **Supplementary Table 1** and **S2**. Detailed genome-build liftOver audit results are provided in **Supplementary Table 3**.

**Table 2:** Instrument selection and harmonization process in the bidirectional Mendelian randomization analyses.

Analysis direction	Exposure variant identification strategy	GWS variants before clumping	Variants with identifiers	Independent variants after LD clumping	Independent variants matched to outcome dataset	Variants retained after harmonization	Final variants included in MR
AIHT to endometriosis	rsID-based selection from the AIHT GWAS	70,287	70,287	274	257	248	248
Endometriosis to AIHT	coordinate-based selection followed by rsID recovery	863	863	27	27	26	26

AIHT, autoimmune hypothyroidism; GRCh37, Genome Reference Consortium Human Build 37; GRCh38, Genome Reference Consortium Human Build 38; GWS, genome-wide significant; LD, linkage disequilibrium; MR, Mendelian randomization; rsID, reference SNP identifier; SNP, single-nucleotide polymorphism.

Genome-wide significant variants were selected using a threshold of P < 5 × 10^-8^, followed by LD clumping using r^2^ < 0.001 within a 10,000-kb window. For the forward analysis, AIHT-associated variants were selected using rsIDs from the AIHT GWAS and converted from GRCh38 to GRCh37 before matching to the endometriosis GWAS. For the reverse analysis, endometriosis-associated variants were initially identified using chromosome, position, and allele information because rsIDs were not directly available in the released summary statistics. These 863 genome-wide significant variants were converted from GRCh37 to GRCh38 and matched to the AIHT reference dataset for rsID recovery before LD clumping. After LD clumping, 27 independent endometriosis instruments were retained; 26 remained after harmonization because one palindromic SNP with intermediate allele frequency was removed according to the default TwoSampleMR harmonization rules.

### Instrument strength, variance explained and power

Instrument strength was assessed using SNP-specific F statistics calculated as β^2^/SE^2^, where β and SE denote the SNP–exposure effect estimate and its standard error, respectively. An F statistic greater than 10 was considered to indicate adequate instrument strength. Approximate SNP-level proportions of variance explained (R^2^) were estimated on the observed binary scale using the relationship R^2^ ≈ F/(F + N − 2), where N denotes the exposure GWAS sample size. Total variance explained was calculated by summing R² values across independent instruments. Statistical power was evaluated using an approximate binary-outcome MR power framework based on outcome sample size, case proportion, total variance explained, alpha level and assumed causal odds ratios (14). Detailed instrument-strength and variance-explained results are provided in **Supplementary Table 1** and **S2**, and power-calculation results are provided in **Supplementary Table 4**.

### Mendelian randomization analyses

The inverse-variance weighted (IVW) method was used as the primary MR approach, in accordance with current recommendations for MR analyses (15, 16). Given the presence of potential heterogeneity across instruments in complex immune-related traits, the primary IVW estimate was obtained using a multiplicative random-effects model. Weighted median and MR-Egger regression were used as complementary methods to evaluate robustness under different assumptions regarding invalid instruments and horizontal pleiotropy (17, 18). Simple mode and weighted mode estimates were additionally generated as supplementary analyses.

**Figure 2:**
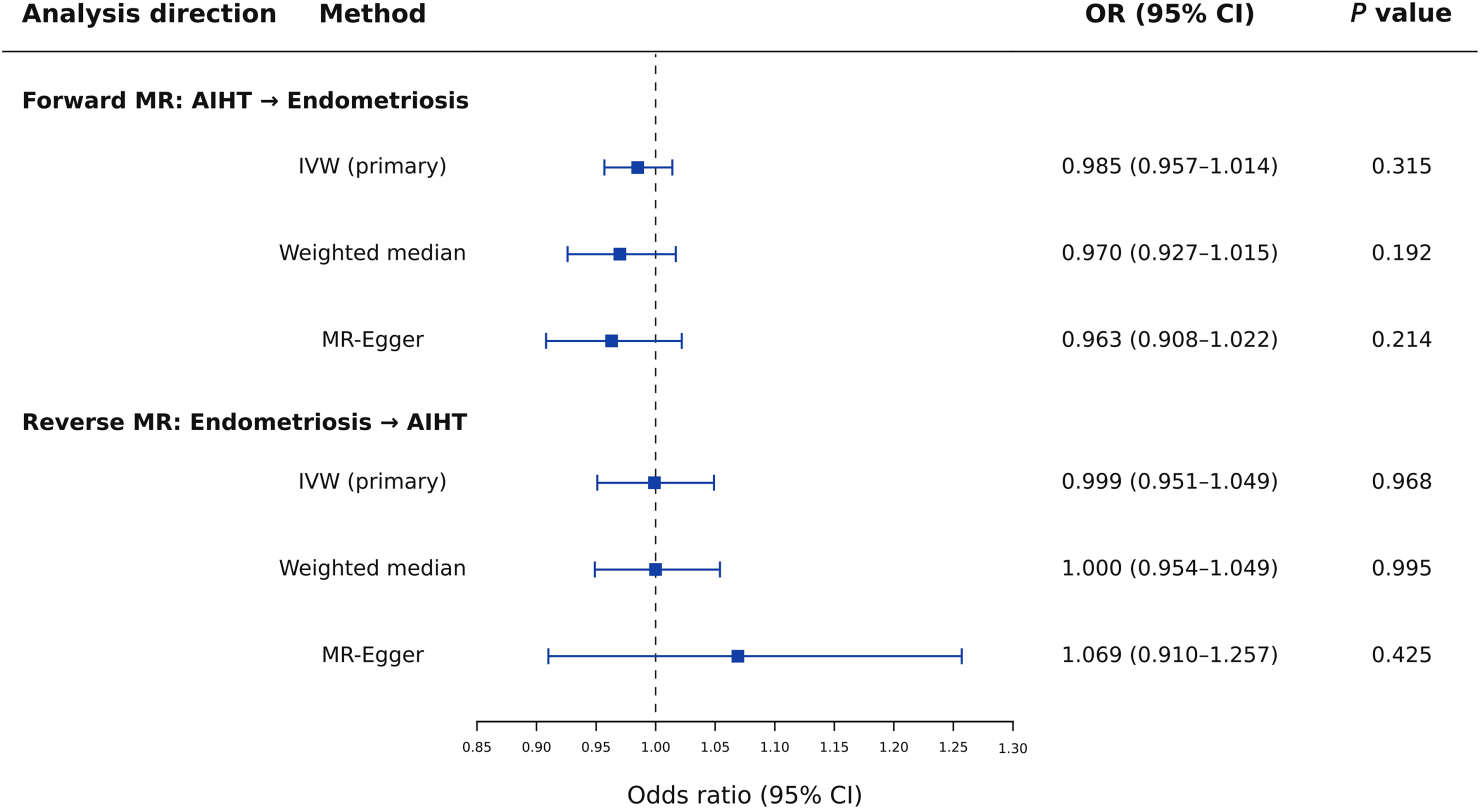
Main bidirectional Mendelian randomization results for autoimmune hypothyroidism and endometriosis.The forest plot shows causal estimates from the bidirectional two-sample Mendelian randomization analyses. The forward analysis evaluated genetic liability to autoimmune hypothyroidism (AIHT) in relation to endometriosis risk, whereas the reverse analysis evaluated genetic liability to endometriosis in relation to AIHT risk. Odds ratios (ORs) and 95% confidence intervals (CIs) are presented for the inverse-variance weighted, weighted median, and MR-Egger methods. No robust evidence of a causal effect was identified in either direction.

MR effect estimates were reported as beta coefficients and converted to odds ratios (ORs) with 95% confidence intervals (CIs). MR estimates were interpreted as the effect of genetic liability to the exposure on the odds of the outcome. The main bidirectional MR results are shown in **Figure 2**, and complete MR estimates are provided in **Supplementary Table 5**.

### Sensitivity analyses

Heterogeneity across instruments was assessed using Cochran’s Q statistic under the IVW and MR-Egger frameworks. Directional horizontal pleiotropy was evaluated using the MR-Egger intercept test. Given the observed between-instrument heterogeneity, the Mendelian randomization pleiotropy residual sum and outlier (MR-PRESSO) method was additionally applied to assess global horizontal pleiotropy and identify potential outlier instruments (19). When a significant outlier was identified, the outlier-corrected estimate and distortion test comparing estimates before and after outlier removal were examined. Complete MR-PRESSO results are provided in **Supplementary Table 6**. Leave-one-out analysis was performed by sequentially removing one SNP at a time and recalculating the IVW estimate to assess whether the overall result was driven by a single influential variant. Detailed leave-one-out results are provided in **Supplementary Table 7**.

To further assess potential violations of the independence and exclusion-restriction assumptions, phenome-wide association study (PheWAS) lookup of harmonized instruments was performed using OpenGWAS. We focused on prespecified trait categories that could plausibly confound or pleiotropically influence the AIHT–endometriosis relationship, including anthropometric, metabolic, lifestyle, socioeconomic, reproductive, endocrine, thyroid-related, autoimmune, inflammatory and pain-related traits.

For the PheWAS-informed review, trait associations reaching genome-wide significance were prioritized for manual evaluation. Variants with relevant PheWAS associations were manually reviewed and classified as “retained after manual review,” “flagged for sensitivity analysis,” or “excluded in sensitivity analysis” before rerunning the sensitivity analyses. Variants without relevant PheWAS associations were retained in the main analysis.

Variants were classified as “retained after manual review” if the associated traits were judged unlikely to influence both AIHT and endometriosis through pathways independent of the exposure. Variants were classified as “flagged for sensitivity analysis” if they were associated with broad immune, inflammatory, endocrine, reproductive, metabolic or pain-related traits that could plausibly indicate pleiotropy but for which the direction or relevance to the AIHT–endometriosis relationship was uncertain. Variants were classified as “excluded in sensitivity analysis” if they were associated with predefined potential confounders or pleiotropic traits that could plausibly affect both the exposure and outcome independent of the target disease pathway.

For AIHT instruments, associations with thyroid-related traits were not automatically considered pleiotropic if they were consistent with the target exposure pathway. PheWAS-informed MR analyses were repeated after excluding variants classified as “excluded in sensitivity analysis,” and a further conservative analysis additionally excluded variants classified as “flagged for sensitivity analysis.”

Because the human leukocyte antigen (HLA) region is highly pleiotropic and central to autoimmune susceptibility, an additional sensitivity analysis was performed after excluding variants located in the extended HLA region. To maintain a consistent coordinate system, HLA-region exclusion was based on GRCh37/hg19 coordinates after genome-build harmonization, with the extended HLA region defined as chromosome 6:25,000,000–34,000,000. Key sensitivity-analysis results are summarized in **Table 3**. Detailed PheWAS lookup and sensitivity-analysis results are provided in **Supplementary Table 8** and **S9**, and HLA-region exclusion results are provided in **Supplementary Table 10**.

**Table 3:** Key sensitivity analyses of the bidirectional Mendelian randomization study.

Analysis direction	Sensitivity analysis	Method/statistic	SNPs (n)	Estimate/value	95% CI/SE	P value
AIHT to endometriosis	Heterogeneity	IVW Cochran’s Q	248	Q = 320.80	df = 247	0.0011
AIHT to endometriosis	Directional pleiotropy	MR-Egger intercept	248	0.0017	SE = 0.0020	0.3885
AIHT to endometriosis	PheWAS-informed analysis 1	IVW OR	146	1.032	0.988–1.077	0.159
AIHT to endometriosis	PheWAS-informed analysis 2	IVW OR	130	1.031	0.985–1.079	0.189
AIHT to endometriosis	HLA-region exclusion analysis	IVW OR	244	0.985	0.955–1.016	0.334
AIHT to endometriosis	MR-PRESSO global test	RSSobs	248	324.14	–	0.0012
Endometriosis to AIHT	Heterogeneity	IVW Cochran’s Q	26	Q = 75.99	df = 25	4.79 × 10^-7^
Endometriosis to AIHT	Directional pleiotropy	MR-Egger intercept	26	-0.0064	SE = 0.0074	0.3956
Endometriosis to AIHT	PheWAS-informed analysis	IVW OR	11	1.028	0.960–1.102	0.429
Endometriosis to AIHT	HLA-region exclusion analysis	IVW OR	26	0.999	0.951–1.049	0.968
Endometriosis to AIHT	MR-PRESSO global test	RSSobs	26	81.77	–	< 2 × 10^-4^
Endometriosis to AIHT	MR-PRESSO outlier-corrected analysis	Corrected OR	25	1.007	0.960–1.056	0.769

AIHT, autoimmune hypothyroidism; CI, confidence interval; df, degrees of freedom; HLA, human leukocyte antigen; IVW, inverse-variance weighted; MR, Mendelian randomization; OR, odds ratio; PheWAS, phenome-wide association study; SE, standard error; SNP, single-nucleotide polymorphism; MR-PRESSO, Mendelian randomization pleiotropy residual sum and outlier; RSSobs, observed residual sum of squares.

PheWAS-informed analysis 1 excluded variants classified as “excluded in sensitivity analysis” after phenome-wide lookup and manual review. PheWAS-informed analysis 2 additionally excluded variants classified as “flagged for sensitivity analysis.” In the reverse analysis, no variants were classified as “flagged for sensitivity analysis,” so only one PheWAS-informed result is shown. The detailed PheWAS-informed, HLA-region exclusion, mode-based, and leave-one-out results are provided in the Supplementary Tables and Figures. MR-PRESSO global tests assessed overall horizontal pleiotropy across the instrumental variables. No individual outlier was identified in the forward analysis. In the reverse analysis, rs2433340 was identified as an outlier; its removal left 25 instruments and yielded an outlier-corrected OR of 1.007 (95% CI: 0.960–1.056, P = 0.769). The distortion test did not indicate a significant difference between the raw and corrected estimates (P = 0.126). Complete MR-PRESSO results are provided in **Supplementary Table 6**.

### Participant-overlap bias assessment

Because the actual participant-overlap fraction between the exposure and outcome GWAS datasets could not be recovered, we performed a scenario-based assessment of overlap-related weak-instrument bias. Following the framework described by Burgess et al. (20), weak-instrument bias in a non-overlapping two-sample analysis is generally expected to be toward the null, whereas increasing participant overlap may progressively shift bias toward the confounded observational association. Under the null causal-effect framework, the relative magnitude of this bias is approximately related to the proportion of participant overlap and inversely related to instrument strength.

The available MR datasets contained SNP-specific F statistics rather than a directly observed study-level first-stage F parameter. We therefore used the minimum observed SNP-specific F statistic in each analysis direction as a deliberately conservative instrument-strength anchor. Assumed participant-overlap proportions of 0%, 10%, 30%, 50% and 100% were evaluated. Conservative approximate relative bias was calculated as p_overlap × (1/F_min) × 100. Because both exposure phenotypes were binary and the exact covariance between SNP–exposure and SNP–outcome association estimates was unavailable, this calculation was interpreted as a scenario-based characterization rather than an exact bias correction. The resulting percentage represents relative weak-instrument bias compared with the bias of the corresponding confounded observational association and does not represent a percentage change in the MR odds ratio. Results are presented in **Supplementary Table 11**.

### Independent clinical cohort analysis

An independent retrospective infertility-clinic cohort was constructed from the Reproductive Medicine Center, Ningbo Women and Children’s Hospital, Ningbo University. The original dataset included 20,781 cycle records from 9,006 women undergoing infertility evaluation with available thyroid function and thyroid autoantibody measurements. Because some women contributed more than one cycle record, the dataset was converted to a patient-level cohort before analysis.

Records were linked using a unique patient identifier. For women with multiple cycle records, the earliest record with complete core thyroid-marker data was retained as the index record. Core thyroid markers included thyroid-stimulating hormone (TSH), free thyroxine (FT4), thyroid peroxidase antibody (TPOAb) and thyroglobulin antibody (TgAb). The thyroid panel test date of the retained record was defined as the index date; these measurements were routinely obtained during infertility evaluation before pregnancy. Baseline covariates and diagnostic information were extracted from the retained index record and the corresponding institutional diagnostic fields. This patient-level conversion was performed to avoid repeated inclusion of the same woman and to ensure that thyroid-related outcomes were assessed using a consistent index record.

Endometriosis status was identified from structured diagnostic records, including primary, additional, and infertility-factor diagnoses. Women were classified as having endometriosis only when endometriosis was recorded as a definite diagnosis in institutional records. According to institutional diagnostic documentation, endometriosis cases included in the analysis were laparoscopically and histopathologically confirmed. Records labeled only as suspected endometriosis were excluded from the primary analysis. Women without a recorded diagnosis of endometriosis during the same infertility-clinic evaluation period were classified as diagnosis-negative controls. Negative laparoscopy was not required for control classification; therefore, clinically unrecognized endometriosis could not be excluded in all controls.

At patient-level screening, 1,049 women were identified from endometriosis-related diagnostic fields and 7,957 women had no recorded endometriosis diagnosis. Exclusions were applied sequentially and are shown separately for the two groups in **Figure 1**. Among women identified from endometriosis-related diagnostic fields, 72 with suspected rather than definite endometriosis, 9 with predefined exclusionary thyroid conditions, 114 with incomplete core thyroid-marker data, and 1 with a missing or unusable thyroid panel date were excluded, leaving 853 women with confirmed endometriosis. Among women without a recorded endometriosis diagnosis, 85 with predefined exclusionary thyroid conditions and 900 with incomplete core thyroid-marker data were excluded, leaving 6,972 diagnosis-negative controls. The final primary clinical analysis set therefore included 7,825 women.

Because the index thyroid measurements were obtained before pregnancy, pregnancy-specific thyroid reference ranges were not applied. Baseline characteristics of the clinical cohort are shown in **Supplementary Table 12**.

### Clinical thyroid outcomes

The primary clinical outcome was AIHT. AIHT was defined as thyroid autoimmunity together with hypothyroidism after excluding major non-autoimmune or treatment-related thyroid conditions. Thyroid autoimmunity was defined as TPOAb positivity and/or TgAb positivity. Serum TPOAb and TgAb were measured using chemiluminescent microparticle immunoassays on the Abbott ARCHITECT i2000 system (Abbott Laboratories). The institutional laboratory reference limits were <5.61 IU/mL for TPOAb and <4.11 IU/mL for TgAb, consistent with the assay-specific manufacturer-recommended reference limits. Accordingly, TPOAb positivity was defined as TPOAb ≥5.61 IU/mL and TgAb positivity as TgAb ≥4.11 IU/mL.

Hypothyroidism was defined as any of the following: elevated TSH above the institutional reference range (TSH >5.91 mIU/L), documented hypothyroidism in diagnostic records, or levothyroxine use consistent with hypothyroid management. Levothyroxine use was considered consistent with hypothyroid management only when it was documented before or at the index thyroid panel date and was accompanied by a recorded diagnosis of hypothyroidism, elevated TSH, or repeated clinical use indicating treatment for hypothyroidism. Levothyroxine use alone was not used to define hypothyroidism if it appeared to represent temporary empirical thyroid optimization in a euthyroid patient without diagnostic or biochemical evidence of hypothyroidism. This rule was applied to reduce misclassification of euthyroid women receiving levothyroxine during infertility care.

Major exclusionary thyroid conditions included Graves’ disease, hyperthyroidism, thyroid cancer, thyroidectomy history and radioiodine treatment history. These exclusions were applied to avoid classifying non-autoimmune, hyperthyroid, malignant, postsurgical or treatment-related thyroid dysfunction as AIHT.

Secondary outcomes included thyroid autoimmunity, TPOAb positivity, TgAb positivity, hypothyroidism, subclinical hypothyroidism and overt hypothyroidism. Subclinical hypothyroidism was defined as TSH >5.91 mIU/L with FT4 within 0.58–1.64 ng/dL, whereas overt hypothyroidism was defined as TSH >5.91 mIU/L with FT4 <0.58 ng/dL. Detailed clinical definitions and reference ranges are provided in **Supplementary Table 13**.

### Clinical cohort statistical analysis

Clinical analyses were conducted in the patient-level primary analysis set. Continuous variables were summarized as mean ± standard deviation or median with interquartile range, as appropriate, and categorical variables as counts and percentages. Logistic regression was used to estimate the association between endometriosis status and thyroid-related outcomes.

Adjusted ORs were estimated using multivariable logistic regression adjusted for age, body mass index (BMI), infertility type, polycystic ovary syndrome (PCOS) and other autoimmune diseases. Covariates were prespecified on clinical and epidemiological grounds rather than selected according to univariable statistical significance. Age and BMI were included because of their relevance to thyroid-related phenotypes and reproductive disease profiles. Infertility type was included to account for differences in reproductive history and clinical ascertainment within an infertility-clinic population. PCOS was included as a common reproductive endocrine disorder associated with distinct infertility and metabolic-endocrine profiles, whereas other autoimmune diseases were included to account for background autoimmune susceptibility relevant to thyroid autoimmunity. The adjusted model was intended to provide a clinically contextualized association estimate rather than a causal effect estimate. Variables that could represent manifestations or closely linked clinical features of established endometriosis were not used for primary covariate selection. Adjusted analyses were performed as complete-case analyses among participants with available data for all prespecified covariates. No imputation of missing covariate data was performed. The main clinical cohort results are shown in **Table 4**.

**Table 4:** Independent clinical cohort analysis results for autoimmune hypothyroidism and thyroid-related outcomes.

Thyroid-related outcome	Diagnosis-negative controls, n/N (%)	Confirmed endometriosis, n/N (%)	Crude OR	95% CI	P value	Adjusted OR	95% CI	P value
Autoimmune hypothyroidism	165/6,972 (2.37)	15/853 (1.76)	0.738	0.433–1.259	0.265	0.753	0.440–1.287	0.299
Thyroid autoimmunity	1,701/6,972 (24.40)	208/853 (24.38)	0.999	0.847–1.179	0.993	1.009	0.854–1.193	0.912
TPOAb positivity	1,046/6,972 (15.00)	118/853 (13.83)	0.910	0.741–1.117	0.365	0.920	0.748–1.132	0.431
TgAb positivity	1,505/6,972 (21.59)	189/853 (22.16)	1.034	0.871–1.227	0.702	1.041	0.875–1.238	0.650
Hypothyroidism	279/6,972 (4.00)	25/853 (2.93)	0.724	0.478–1.097	0.128	0.760	0.500–1.155	0.198

AIHT, autoimmune hypothyroidism; BMI, body mass index; CI, confidence interval; OR, odds ratio; PCOS, polycystic ovary syndrome; TgAb, thyroglobulin antibody; TPOAb, thyroid peroxidase antibody.

The independent clinical cohort was analyzed using the patient-level primary analysis set. Crude ORs were estimated using univariable logistic regression. Adjusted ORs were estimated using multivariable logistic regression adjusted for age, BMI, infertility type, PCOS, and other autoimmune diseases. Adjusted analyses were based on 7,770 participants with complete covariate data. Autoimmune hypothyroidism was defined as thyroid autoimmunity plus hypothyroidism after excluding Graves’ disease, hyperthyroidism, thyroid cancer, thyroidectomy history, and radioiodine treatment history. Thyroid autoimmunity was defined as TPOAb positivity and/or TgAb positivity.

Sensitivity analyses were performed by excluding women with PCOS, excluding women with other autoimmune diseases, excluding women with adenomyosis and applying a stricter AIHT definition requiring thyroid autoimmunity plus elevated TSH. Detailed clinical sensitivity analyses are provided in **Supplementary Table 14**.

### Software and ethics

All MR analyses were conducted using R version 4.5.1 with the data.table, dplyr, TwoSampleMR, ieugwasr, GenomicRanges, IRanges and rtracklayer packages (9, 12, 13). Clinical cohort analyses were also performed in R. All statistical tests were two-sided, and P < 0.05 was considered nominally statistically significant. Analyses of secondary thyroid-related outcomes and sensitivity analyses were interpreted as exploratory and were assessed based on consistency of effect direction, effect size, precision and robustness across models rather than statistical significance alone.

The MR analyses used publicly available, de-identified GWAS summary statistics from previously published studies. The independent clinical cohort analysis used de-identified clinical data from infertility-clinic records and was approved by the Ethics Committee of Ningbo Women and Children’s Hospital, Ningbo University (NBFE-2026-KY-081). The requirement for individual informed consent was waived because of the retrospective design and use of de-identified data.

## Results

### Instrument selection and strength

The overall study workflow is shown in **Figure 1**, and the GWAS and clinical data sources are summarized in **Table 1**. After instrument selection, genome-build harmonization, outcome matching, and allele harmonization, 248 SNPs were retained for the AIHT-to-endometriosis analysis and 26 SNPs for the endometriosis-to-AIHT analysis. The full instrument selection and harmonization process is summarized in **Table 2**.

All retained instruments showed adequate strength. In the AIHT-to-endometriosis analysis, SNP-specific F statistics ranged from 29.70 to 1,422.48, with a median of 52.88 and a mean of 96.55. In the endometriosis-to-AIHT analysis, F statistics ranged from 30.27 to 115.85, with a median of 37.53 and a mean of 47.37. The 248 AIHT instruments explained approximately 2.94% of the observed-scale variance in the binary AIHT phenotype, whereas the 26 endometriosis instruments explained approximately 0.26% of the observed-scale variance in the binary endometriosis phenotype. Detailed instrument characteristics and variance-explained estimates are provided in **Supplementary Table 1** and **S2**. Power calculations indicated greater statistical power in the forward analysis. The approximate minimum detectable OR at 80% power was 1.118 for the AIHT-to-endometriosis analysis and 1.224 for the endometriosis-to-AIHT analysis, indicating limited power to exclude small reverse-direction effects. Detailed power estimates are provided in **Supplementary Table 4**.

### Main bidirectional MR results

The main bidirectional MR results are shown in **Figure 2**. In the forward analysis, genetic liability to AIHT was not associated with endometriosis risk using the primary multiplicative random-effects IVW method (OR = 0.985, 95% CI: 0.957–1.014, P = 0.315). The weighted median estimate was OR = 0.970 (95% CI: 0.927–1.015, P = 0.192), and the MR-Egger estimate was OR = 0.963 (95% CI: 0.908–1.022, P = 0.214). Thus, none of the three principal MR estimators provided evidence of a non-null association in the forward direction.

In the reverse analysis, the IVW estimate was close to the null (OR = 0.999, 95% CI: 0.951–1.049, P = 0.968). The weighted median estimate was OR = 1.000 (95% CI: 0.954–1.049, P = 0.995), and the MR-Egger estimate was OR = 1.069 (95% CI: 0.910–1.257, P = 0.425). None of the three principal estimators provided evidence of a non-null association, although the wider CI of the MR-Egger estimate indicated greater imprecision in the reverse analysis. Complete bidirectional MR estimates, including simple mode and weighted mode analyses, are provided in **Supplementary Table 5**, and the corresponding scatter plots are shown in **Supplementary Figure 1** and **S2**.

### MR sensitivity analyses

Selected MR sensitivity analyses are summarized in **Table 3**. Cochran’s Q indicated significant heterogeneity across instruments in both the AIHT-to-endometriosis analysis (IVW Cochran’s Q = 320.80, degrees of freedom (df) = 247, P = 0.0011) and the endometriosis-to-AIHT analysis (IVW Cochran’s Q = 75.99, df = 25, P = 4.79 × 10^-7^). MR-Egger intercept tests provided no statistical evidence of directional horizontal pleiotropy in either the forward analysis (intercept = 0.0017, SE = 0.0020, P = 0.3885) or the reverse analysis (intercept = −0.0064, SE = 0.0074, P = 0.3956). The corresponding funnel plots are shown in **Supplementary Figure 3** and **S4**.

MR-PRESSO provided additional evidence of horizontal pleiotropy at the global level. In the forward AIHT-to-endometriosis analysis, the global test was significant, with an observed residual sum of squares (RSSobs) of 324.14 (P = 0.0012), but no individual outlier instrument was identified; therefore, no outlier-corrected estimate was available. In the reverse endometriosis-to-AIHT analysis, the global test was also significant (RSSobs = 81.77, P < 2 × 10^-4^) and identified rs2433340 as an outlier. After removal of rs2433340, the estimate remained close to the null and non-significant (OR = 1.007, 95% CI: 0.960–1.056, P = 0.769). The distortion test did not indicate a significant change between the raw and outlier-corrected estimates (P = 0.126). Complete MR-PRESSO results are provided in **Supplementary Table 6**.

Leave-one-out analyses showed that sequential removal of individual SNPs did not materially alter the overall IVW estimates. Detailed leave-one-out results are provided in **Supplementary Table 7**, and the corresponding leave-one-out plots are shown in **Supplementary Figure 5** and **S6**. Single-SNP forest plots for the forward and reverse analyses are provided in **Supplementary Figure 7** and **S8**, respectively.

After PheWAS-informed exclusion of potentially pleiotropic variants, the forward IVW point estimate crossed the null but remained close to 1. Exclusion of 102 variants classified as “excluded in sensitivity analysis” yielded an IVW OR of 1.032 (95% CI: 0.988–1.077, P = 0.159), whereas additional exclusion of 16 variants classified as “flagged for sensitivity analysis” yielded an IVW OR of 1.031 (95% CI: 0.985–1.079, P = 0.189). Among the 102 variants removed in the first analysis, PheWAS associations predominantly involved anthropometric/metabolic and autoimmune/inflammatory traits, with overlapping thyroid-related, AIHT-related, reproductive/endocrine, lifestyle/socioeconomic, and pain-related categories. These categories were not mutually exclusive. The additional 16 flagged variants had broad autoimmune/inflammatory associations.

Forward IVW heterogeneity was substantially reduced after PheWAS-informed filtering, from Q = 320.80 (df = 247, P = 0.0011) in the primary analysis to Q = 153.84 (df = 145, P = 0.292) after exclusion of the 102 classified variants and Q = 139.42 (df = 129, P = 0.250) after the additional exclusion of the 16 flagged variants. In the two filtered analyses, weighted median estimates were nominally associated with increased endometriosis odds (OR = 1.076, 95% CI: 1.007–1.150, P = 0.031; and OR = 1.076, 95% CI: 1.006–1.150, P = 0.033, respectively), whereas the corresponding IVW and MR-Egger estimates remained non-significant.

In the reverse analysis, exclusion of 15 variants classified as “excluded in sensitivity analysis” left 11 instruments. The IVW estimate remained non-significant (OR = 1.028, 95% CI: 0.960–1.102, P = 0.429). No additional reverse-direction variants were classified as “flagged for sensitivity analysis.” Detailed variant classifications, diagnostic results, and MR estimates are provided in **Supplementary Table 8** and **S9**.

After excluding variants located in the extended HLA region, the forward IVW estimate remained consistent with the primary analysis (244 SNPs; OR = 0.985, 95% CI: 0.955–1.016, P = 0.334). No HLA-region variants were identified among the endometriosis instruments in the reverse analysis; therefore, the reverse HLA-region exclusion estimate was identical to the primary reverse analysis (26 SNPs; OR = 0.999, 95% CI: 0.951–1.049, P = 0.968). Detailed HLA-region sensitivity results are provided in **Supplementary Table 10**.

In the scenario-based assessment of potential participant-overlap bias, an assumed 30% overlap corresponded to approximate relative bias of 1.01% and 0.99% in the forward and reverse analyses, respectively. The corresponding estimates were 1.68% and 1.65% at 50% assumed overlap and 3.37% and 3.30% under a hypothetical complete-overlap scenario. These scenario-based estimates were small relative to the bias of the corresponding confounded observational association, although the actual overlap fraction and the numerical direction of bias could not be determined. Complete scenario results are presented in **Supplementary Table 11**.

### Independent clinical cohort results

The independent clinical cohort was derived from 9,006 women undergoing infertility evaluation. After the sequential patient-level exclusions shown in **Figure 1**, the primary clinical analysis set comprised 7,825 women, including 853 women with confirmed endometriosis and 6,972 diagnosis-negative controls. Baseline characteristics of the clinical cohort are presented in **Supplementary Table 12**. Compared with controls, women with endometriosis were slightly younger, had lower BMI, and more frequently had adenomyosis and pelvic adhesion. Among the 7,825 women in the primary clinical analysis set, **55** had missing data for at least one prespecified adjustment covariate and were excluded from the complete-case multivariable analyses, leaving 7,770 participants in the adjusted models.

The clinical cohort results are summarized in **Table 4**. The prevalence of AIHT was 1.76% in women with endometriosis and 2.37% in controls. Endometriosis was not associated with AIHT in either crude analysis or multivariable logistic regression adjusted for age, BMI, infertility type, PCOS, and other autoimmune diseases (adjusted OR = 0.753, 95% CI: 0.440–1.287, P = 0.299).

The prevalence of thyroid autoimmunity was similar between women with and without endometriosis (24.38% vs 24.40%), and the adjusted estimate was close to the null (adjusted OR = 1.009, 95% CI: 0.854–1.193, P = 0.912). Similarly, no association was observed for TPOAb positivity (adjusted OR = 0.920, 95% CI: 0.748–1.132, P = 0.431), TgAb positivity (adjusted OR = 1.041, 95% CI: 0.875–1.238, P = 0.650), or hypothyroidism (adjusted OR = 0.760, 95% CI: 0.500–1.155, P = 0.198).

Sensitivity analyses excluding women with PCOS, excluding women with other autoimmune diseases, excluding women with adenomyosis, and applying a stricter AIHT definition requiring thyroid autoimmunity plus elevated TSH did not materially change the clinical cohort findings. Thyroid outcome definitions and reference ranges are provided in **Supplementary Table 13**, and detailed clinical sensitivity analyses are provided in **Supplementary Table 14**.

## Discussion

In this bidirectional MR study complemented by an independent clinical cohort analysis, we found no robust evidence supporting a causal relationship between AIHT and endometriosis in either direction. Genetic liability to AIHT was not associated with endometriosis risk, and genetic liability to endometriosis was not associated with AIHT risk. The main MR estimates remained close to the null across complementary estimators and sensitivity analyses, although significant between-instrument heterogeneity and evidence from MR-PRESSO global tests warrant cautious interpretation. In parallel, the independent infertility-clinic cohort showed no clear association between endometriosis and AIHT or thyroid autoimmunity. Together, the genetic and clinical analyses yielded broadly concordant null findings, but these findings should not be interpreted as establishing the absence of a causal effect or clinical association (15, 16).

The absence of robust causal evidence should be interpreted in the context of the known biological and clinical overlap between endometriosis and immune-mediated disorders. Endometriosis is increasingly recognized as a systemic gynecological disorder involving inflammatory activation, immune dysregulation, hormonal dependence, and genetic susceptibility (1, 2, 21). Recent genetic and epidemiological studies have also suggested phenotypic or genetic overlap between endometriosis and selected immune-mediated conditions (4, 22). AIHT, in turn, is a prototypic organ-specific autoimmune endocrine disease and is clinically relevant to women’s reproductive health (3, 23, 24). Therefore, a relationship between thyroid autoimmunity and endometriosis is biologically plausible. However, biological plausibility and clinical co-occurrence do not necessarily imply direct causality. Shared immune-inflammatory susceptibility, diagnostic surveillance, reproductive health-seeking behavior and residual confounding remain possible explanations for previously reported clinical overlap. However, the present analyses cannot determine which, if any, of these mechanisms accounts for the observed comorbidity reported in previous studies.

This study also refines the interpretation of previous thyroid-related reproductive MR studies. Prior MR work has mainly evaluated thyroid function biomarkers, such as thyrotropin and free thyroxine, or broad hypothyroidism or hyperthyroidism phenotypes (5, 6). These exposures may capture circulating hormone regulation or heterogeneous thyroid dysfunction rather than autoimmune thyroid disease itself. By using a recently characterized disease-specific AIHT GWAS, the present study focused more directly on autoimmune thyroid dysfunction (3). The null findings suggest that genetic liability to AIHT is unlikely to be a major determinant of overall endometriosis susceptibility. Nevertheless, this does not exclude the possibility that thyroid autoimmunity or thyroid dysfunction may be relevant to other reproductive outcomes, including implantation, miscarriage, ovarian reserve, and assisted reproduction outcomes (23–25), which were not evaluated in the present MR analysis.

The independent clinical cohort analysis provided a complementary clinical perspective. In this infertility-clinic population, endometriosis was not associated with a higher prevalence of AIHT or thyroid autoimmunity after adjustment for relevant clinical covariates. This observation is clinically informative because infertility patients commonly undergo systematic thyroid screening, which may reduce some forms of differential ascertainment compared with general observational settings. The broadly concordant null results from the MR and clinical analyses do not provide clear support for a strong AIHT–endometriosis association.

The sensitivity analyses further support a cautious interpretation of the MR results. The selected instruments showed adequate strength, and the forward analysis had greater statistical power than the reverse analysis because the AIHT instruments explained a larger proportion of exposure variance. This imbalance is important. The approximate minimum detectable OR at 80% power was 1.118 in the forward analysis and 1.224 in the reverse analysis. Therefore, the absence of evidence for an effect of genetic liability to endometriosis on AIHT should not be interpreted as evidence that no small reverse-direction effect exists; the reverse analysis was primarily informative for moderate or larger effects under the assumptions of the power calculation.

Significant between-instrument heterogeneity was observed in both directions and limits the interpretation of any single pooled IVW estimate in isolation. The multiplicative random-effects IVW model used in the primary analysis allows for overdispersion when estimating uncertainty but does not eliminate the underlying sources of heterogeneity or exclude horizontal pleiotropy. We therefore interpreted the IVW results together with the weighted median and MR-Egger estimates, which rely on different assumptions regarding invalid instruments. None of these principal estimators consistently supported a non-null association in either direction. MR-PRESSO global tests were significant in both directions, providing additional evidence of pleiotropic heterogeneity. No individual outlier was identified in the forward analysis. In the reverse analysis, rs2433340 was identified as an outlier, but its removal yielded an estimate that remained close to the null, and the distortion test did not indicate a significant change from the raw estimate. Thus, the MR-PRESSO findings did not materially alter the overall null interpretation, although they reinforce the need for caution regarding between-instrument heterogeneity and potential horizontal pleiotropy. The absence of a significant MR-Egger intercept indicates no statistical evidence of directional horizontal pleiotropy but does not exclude balanced pleiotropy or other sources of between-instrument heterogeneity.

The crossing of the null by the forward IVW point estimate after PheWAS-informed filtering also warrants cautious interpretation. Both the primary IVW estimate and the filtered IVW estimates were close to the null and had CIs that included 1; therefore, this change should not be interpreted as a confirmed reversal from a protective to a risk effect. The excluded variants predominantly showed anthropometric/metabolic and autoimmune/inflammatory associations, with overlapping thyroid-related, AIHT-related, reproductive/endocrine, lifestyle/socioeconomic, and pain-related associations. The marked reduction in Cochran’s Q after PheWAS-informed filtering suggests that these variants contributed to between-instrument heterogeneity. Balanced horizontal pleiotropy remains a possible contributor to this heterogeneity and cannot be excluded by the non-significant MR-Egger intercept. However, the filtered analyses did not yield concordant evidence across MR estimators: weighted median estimates were nominally significant, whereas the corresponding IVW and MR-Egger estimates remained non-significant. We therefore regard the weighted median findings as exploratory, method-specific signals rather than robust evidence of a causal effect.

This study has several strengths. First, the bidirectional MR design allowed assessment of both AIHT-to-endometriosis and endometriosis-to-AIHT directions, reducing the risk of interpreting clinical comorbidity as a unidirectional causal relationship. Second, the exposure definition focused on disease-specific AIHT rather than relying only on thyroid function biomarkers or broad hypothyroidism definitions, improving biological specificity for autoimmune thyroid dysfunction (3). Third, the MR analysis incorporated stringent instrument selection and extensive sensitivity analyses addressing instrument strength, heterogeneity, potential pleiotropy, and HLA-region effects. Fourth, the study included an independent clinical cohort of infertile women, allowing the genetic findings to be interpreted alongside real-world thyroid autoimmunity and hypothyroidism phenotypes.

Several limitations should be acknowledged. First, the AIHT and endometriosis GWAS datasets were derived mainly from European ancestry populations, whereas the clinical cohort consisted of Chinese infertility-clinic patients. This difference limits direct comparability between the genetic and clinical components and may affect generalizability across ancestries.

Second, participant overlap between the AIHT and endometriosis GWAS datasets could not be quantified directly. Under the conservative scenario-based assessment using the minimum observed SNP-specific F statistics, assumed overlap of 30% corresponded to approximately 1.0% relative bias and 50% overlap to approximately 1.7% relative bias in both analysis directions. These values are theoretical relative-bias scenarios rather than corrections of the reported ORs, and the actual direction and magnitude of overlap-related bias could not be established because the true overlap fraction and the direction of the confounded association in the overlapping source samples were unavailable.

Third, the AIHT GWAS was sex-combined. In the reverse analysis, SNP–endometriosis associations were derived exclusively from women, whereas SNP–AIHT associations were estimated in a population including both women and men. Although sex was included in the original AIHT GWAS models, covariate adjustment for sex does not provide female-specific genetic effect estimates or exclude sex heterogeneity in SNP–AIHT associations. The reverse MR estimate should therefore not be interpreted as a strictly women-specific causal estimate.

Fourth, endometriosis was evaluated as an overall disease phenotype in the MR analysis. We could not examine lesion subtype, disease stage, pain-dominant disease, ovarian endometrioma, deep infiltrating endometriosis, or infertility-associated endometriosis. Future genetically informed and clinically phenotyped studies should prioritize subgroup analyses of ovarian endometrioma, deep infiltrating endometriosis, and superficial peritoneal endometriosis, as well as stage-stratified and infertility-associated disease, because associations with thyroid autoimmunity may differ across clinically and anatomically distinct endometriosis phenotypes.

Fifth, both MR exposures were based on genetic liability to binary disease phenotypes and therefore did not capture disease duration, thyroid antibody titers, thyroid hormone dynamics, treatment exposure, inflammatory activity, or changes over the reproductive life course (15). Sixth, the modest variance explained by the endometriosis instruments limited the ability of the reverse MR analysis to exclude small effects. Finally, residual pleiotropy cannot be fully excluded, particularly for immune-related traits with shared genetic architecture.

The clinical cohort analysis also has limitations. Its retrospective and cross-sectional nature precluded assessment of the temporal sequence between endometriosis and thyroid-related outcomes. Accordingly, this component of the study can assess cross-sectional associations and clinical comorbidity patterns but cannot establish whether endometriosis preceded thyroid autoimmunity or AIHT, whether thyroid disease preceded endometriosis, or whether both conditions arose from shared background factors. The clinical cohort was intended to provide clinical contextualization of the MR findings rather than an independent test of causality; causal inference in the present study is derived from the MR component, subject to its underlying assumptions and limitations.

The cohort was derived from an infertility-clinic population, which improves relevance to reproductive medicine but limits direct generalizability to non-infertile women and population-based samples. The reproductive disease spectrum and clinical ascertainment in this setting may preferentially capture clinically recognized or infertility-associated endometriosis, whereas milder, asymptomatic, or non-infertility-associated disease may be underrepresented. Therefore, the absence of a clear association between endometriosis and AIHT or thyroid autoimmunity in this cohort should be interpreted within the infertility-clinic context and does not establish that no association exists across all endometriosis populations.

Although endometriosis cases were identified from institutional diagnostic records and were documented as laparoscopically and histopathologically confirmed, the controls were diagnosis-negative rather than laparoscopically confirmed to be free of endometriosis. Asymptomatic, minimally symptomatic, or otherwise clinically unrecognized endometriosis may therefore have been present among some controls. If this misclassification was largely nondifferential with respect to thyroid status, contamination of the control group would be expected to attenuate between-group differences toward the null and could reduce the ability to detect a modest association. However, because differential clinical ascertainment cannot be fully excluded, the direction of bias cannot be determined with certainty. Similarly, AIHT classification relied on available thyroid function tests, thyroid autoantibodies, diagnostic records, and levothyroxine-use information. Levothyroxine treatment may normalize contemporaneous TSH concentrations and thereby obscure the untreated biochemical phenotype in some women. Because levothyroxine use represents disease management downstream of thyroid disease recognition and was partly incorporated into the clinical classification of AIHT or hypothyroidism when consistent with hypothyroid treatment, it was not included as a conventional adjustment covariate. Nevertheless, although exclusionary thyroid conditions were removed and a stricter AIHT definition was examined in sensitivity analysis, residual treatment-related classification uncertainty and other sources of misclassification cannot be completely excluded. Population-specific thyroid autoantibody reference intervals were not independently re-established within this infertility cohort, and residual threshold-related classification uncertainty therefore remains possible. Finally, residual confounding by unmeasured factors, such as disease duration, prior surgery, inflammatory burden, family history, iodine status, or detailed reproductive treatment history, could not be fully addressed.

These analyses do not address the broader clinical indications for thyroid assessment in infertility, miscarriage, or assisted reproduction, where thyroid dysfunction and thyroid autoimmunity remain clinically relevant (23, 24). Accordingly, the present results should not be used to reduce clinically indicated thyroid evaluation; rather, they suggest that a causal AIHT–endometriosis relationship should not be assumed solely on the basis of reported clinical comorbidity.

Larger GWAS datasets with subtype-specific endometriosis definitions, thyroid antibody phenotypes, and broader ancestry representation would improve causal inference. Prospective cohorts integrating thyroid autoantibody status, thyroid hormone trajectories, immune profiling, endometriosis subtype, and assisted reproductive outcomes may help determine whether thyroid autoimmunity modifies fertility prognosis or treatment response among women with endometriosis. Experimental and translational studies may also clarify whether shared immune-inflammatory pathways contribute to the coexistence of these conditions without implying a direct causal pathway.

In conclusion, this bidirectional MR study, complemented by an independent infertility-clinic cohort analysis, did not provide robust evidence that genetic liability to AIHT materially affects overall endometriosis risk or that genetic liability to endometriosis materially affects AIHT risk in the datasets analyzed. The clinical cohort similarly showed no clear association between diagnosed endometriosis and AIHT or thyroid autoimmunity. However, potential participant overlap between GWAS datasets, the use of sex-combined AIHT summary statistics, limited power to exclude small reverse-direction effects, and possible clinically unrecognized endometriosis among diagnosis-negative controls warrant cautious interpretation. Modest effects and sex-specific or endometriosis-subtype-specific associations cannot be excluded.

## Data Availability

AIHT GWAS summary statistics were obtained from the FinnGen R12–UK Biobank GWAS meta-analysis reported by Reeve et al. using phenotype E4_HYTHY_AI_STRICT. FinnGen R12 data-access information is available at https://finngen.gitbook.io/documentation/data-download, and the R12 manifest is available at https://storage.googleapis.com/finngen-public-data-r12/summary_stats/finngen_R12_manifest.tsv. Endometriosis GWAS summary statistics were obtained from the NHGRI-EBI GWAS Catalog, accession GCST90205183: https://www.ebi.ac.uk/gwas/studies/GCST90205183. The de-identified clinical validation dataset is restricted by institutional privacy and ethical requirements. R scripts are provided as **Supplementary File 1**.

## References

[1] Rahmioglu N Mortlock S Ghiasi M Møller PL Stefansdottir L Galarneau G . The genetic basis of endometriosis and comorbidity with other pain and inflammatory conditions. Nat Genet. (2023) 55:423–36. doi: 10.1038/s41588-023-01323-z 36914876 PMC10042257

[2] Garvey M . Endometriosis: Future biological perspectives for diagnosis and treatment. Int J Mol Sci. (2024) 25:12242. doi: 10.3390/ijms252212242 39596309 PMC11595046

[3] Reeve MP Kanai M Graham D Karjalainen J Luo S Kolosov N . Genome-wide association analyses of autoimmune hypothyroidism reveal autoimmune and thyroid-specific contributions and an inverse relationship with cancer risk. Nat Genet. (2026) 58:550–9. doi: 10.1038/s41588-026-02521-1 41748903 PMC12987720

[4] Shigesi N Harris HR Fang H Ndungu A Lincoln MR Cotsapas C . The phenotypic and genetic association between endometriosis and immunological diseases. Hum Reprod. (2025) 40:1195–209. doi: 10.1093/humrep/deaf062 40262193 PMC12127507

[5] Liu Q Qiu Y Jiang J Long S Zhu C Chen G . Causal association between thyroid function and the risk of infertility: a Mendelian randomization study. Front Endocrinol. (2024) 15:1425639. doi: 10.3389/fendo.2024.1425639 39429737 PMC11486735

[6] Liang Z Xu Z Liu J . Mendelian randomization study of thyroid function and anti-Müllerian hormone levels. Front Endocrinol. (2023) 14:1188284. doi: 10.3389/fendo.2023.1188284 37547307 PMC10400324

[7] Davies NM Holmes MV Davey Smith G . Reading Mendelian randomisation studies: a guide, glossary, and checklist for clinicians. BMJ. (2018) 362:k601. doi: 10.1136/bmj.k601 30002074 PMC6041728

[8] FinnGen . Finngen Public Documentation: Data Releases and R12 Summary Statistics. Available online at: https://finngen.gitbook.io/documentation/ (Accessed April 23, 2026).

[9] Hemani G Zheng J Elsworth B Wade KH Haberland V Baird D . The MR-Base platform supports systematic causal inference across the human phenome. Elife. (2018) 7:e34408. doi: 10.7554/eLife.34408 29846171 PMC5976434

[10] Zhang W Liu E Que H . Association of circulating vitamin levels with thyroid diseases: a Mendelian randomization study. Front Endocrinol. (2024) 15:1360851. doi: 10.3389/fendo.2024.1360851 38919472 PMC11196410

[11] Lu FF Wang Z Yang QQ Yan FS Xu C Wang MT . Investigating the metabolomic pathways in female reproductive endocrine disorders: a Mendelian randomization study. Front Endocrinol. (2024) 15:1438079. doi: 10.3389/fendo.2024.1438079 39544240 PMC11560792

[12] Hinrichs AS Karolchik D Baertsch R Barber GP Bejerano G Clawson H . The UCSC genome browser database: update 2006. Nucleic Acids Res. (2006) 34:D590–8. doi: 10.1093/nar/gkj144 16381938 PMC1347506

[13] Lawrence M Gentleman R Carey V . rtracklayer: an R package for interfacing with genome browsers. Bioinformatics. (2009) 25:1841–2. doi: 10.1093/bioinformatics/btp328 19468054 PMC2705236

[14] Burgess S . Sample size and power calculations in Mendelian randomization with a single instrumental variable and a binary outcome. Int J Epidemiol. (2014) 43:922–9. doi: 10.1093/ije/dyu005 24608958 PMC4052137

[15] Burgess S Woolf B Mason AM Ala-Korpela M Gill D . Addressing the credibility crisis in Mendelian randomization. BMC Med. (2024) 22:374. doi: 10.1186/s12916-024-03607-5 39256834 PMC11389083

[16] Burgess S Davey Smith G Davies NM Dudbridge F Gill D Glymour MM . Guidelines for performing Mendelian randomization investigations: update for summer 2023. Wellcome Open Res. (2023) 4:186. doi: 10.12688/wellcomeopenres.15555.3 32760811 PMC7384151

[17] Bowden J Davey Smith G Haycock PC Burgess S . Consistent estimation in Mendelian randomization with some invalid instruments using a weighted median estimator. Genet Epidemiol. (2016) 40:304–14. doi: 10.1002/gepi.21965 27061298 PMC4849733

[18] Bowden J Davey Smith G Burgess S . Mendelian randomization with invalid instruments: effect estimation and bias detection through Egger regression. Int J Epidemiol. (2015) 44:512–25. doi: 10.1093/ije/dyv080 26050253 PMC4469799

[19] Verbanck M Chen CY Neale B Do R . Detection of widespread horizontal pleiotropy in causal relationships inferred from Mendelian randomization between complex traits and diseases. Nat Genet. (2018) 50:693–8. doi: 10.1038/s41588-018-0099-7 29686387 PMC6083837

[20] Burgess S Davies NM Thompson SG . Bias due to participant overlap in two-sample Mendelian randomization. Genet Epidemiol. (2016) 40:597–608. doi: 10.1002/gepi.21998 27625185 PMC5082560

[21] Shifon S Tyrinova T Veretelnikova T Pasman N Chernykh E . Endometriosis as an immune-mediated disease: pathogenetic mechanisms and therapeutic strategies. Front Immunol. (2025) 16:1727183. doi: 10.3389/fimmu.2025.1727183 41488658 PMC12756115

[22] Aziz M Beaton MA Aziz MA Opoku-Anane J Elhadad N . Endometriosis and autoimmunity: a large-scale case-control study of endometriosis and 10 distinct autoimmune diseases. NPJ Womens Health. (2025) 3:36. doi: 10.1038/s44294-025-00086-8 40547362 PMC12176652

[23] Popa EC Maghiar L Maghiar TA Brihan I Georgescu LM Toderaș BA . Hashimoto’s thyroiditis and female fertility: endocrine, immune, and microbiota perspectives in assisted reproduction: a narrative review. Biomedicines. (2025) 13:1495. doi: 10.3390/biomedicines13061495 40564214 PMC12190467

[24] Huang Y Xie B Li J Hang F Hu Q Jin Y . Prevalence of thyroid autoantibody positivity in women with infertility: a systematic review and meta-analysis. BMC Womens Health. (2024) 24:630. doi: 10.1186/s12905-024-03473-6 39604908 PMC11600930

[25] Tan J Yang YY Yin DY Xin Q Ge XC . Research on the impact of thyroid disorders on reproductive function: a narrative review. J Clin Med Res. (2025) 17:409–22. doi: 10.14740/jocmr6315 40958985 PMC12434935

