# Supplementary material for: Autoimmune hypothyroidism and endometriosis: evidence from bidirectional Mendelian randomization and an independent clinical cohort analysis
Source: Front Endocrinol (Lausanne). 2026 Aug 4;17:1898299. doi: 10.3389/fendo.2026.1898299 (PMC13487738; doi:10.3389/fendo.2026.1898299)
Supplement: Supplementary file 1 [file DataSheet1.zip › Supporting information/S3_Forward funnel plot.pdf]

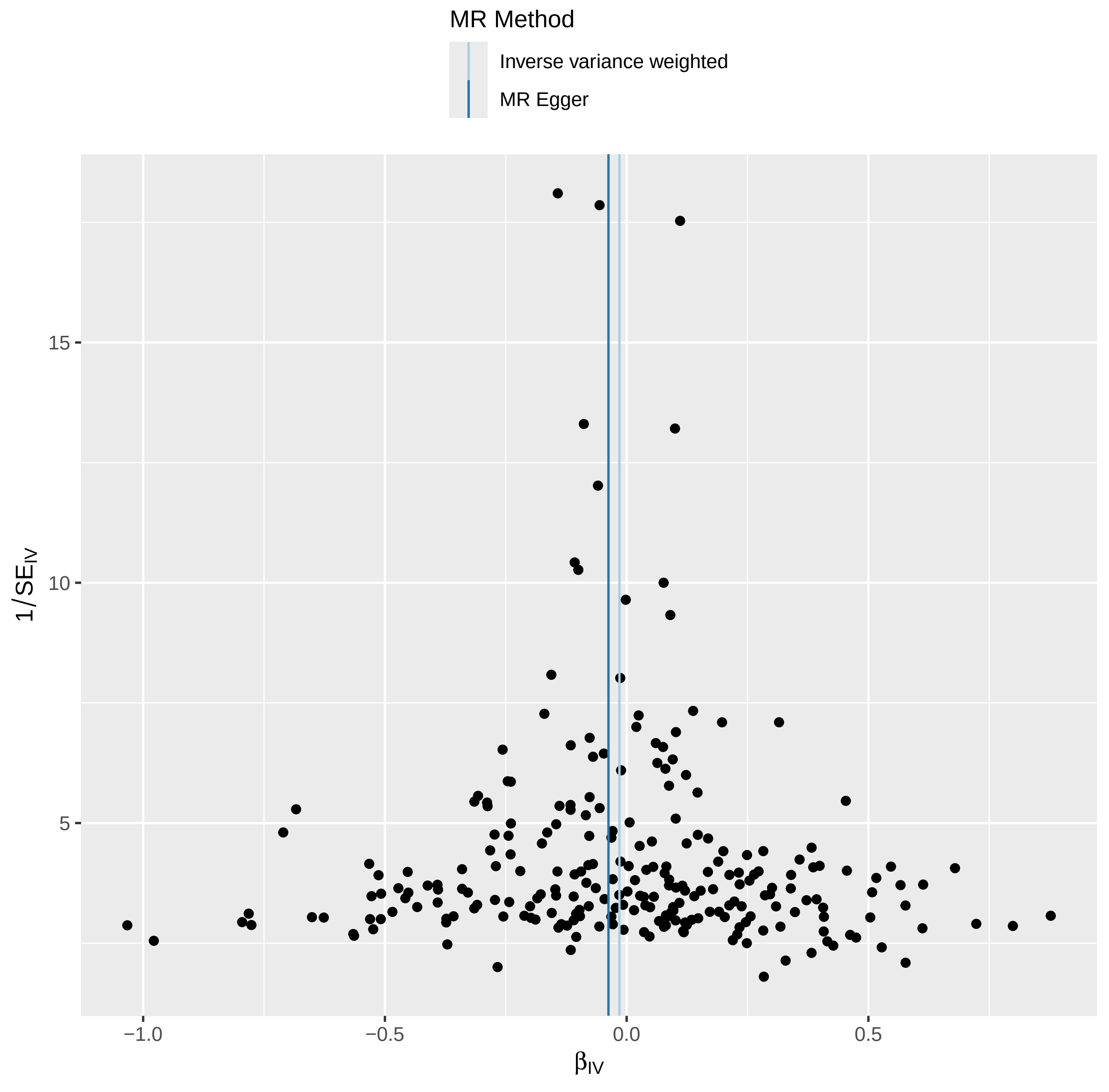

MR Method
Inverse variance weighted
MR Egger
15
10
5
1/SE$_{IV}$
−1.0
−0.5
0.0
0.5
β$_{IV}$
