# Supplementary material for: Autoimmune hypothyroidism and endometriosis: evidence from bidirectional Mendelian randomization and an independent clinical cohort analysis
Source: Front Endocrinol (Lausanne). 2026 Aug 4;17:1898299. doi: 10.3389/fendo.2026.1898299 (PMC13487738; doi:10.3389/fendo.2026.1898299)
Supplement: Supplementary file 1 [file DataSheet1.zip › Supporting information/S5_Forward leave-one-out plot.pdf]

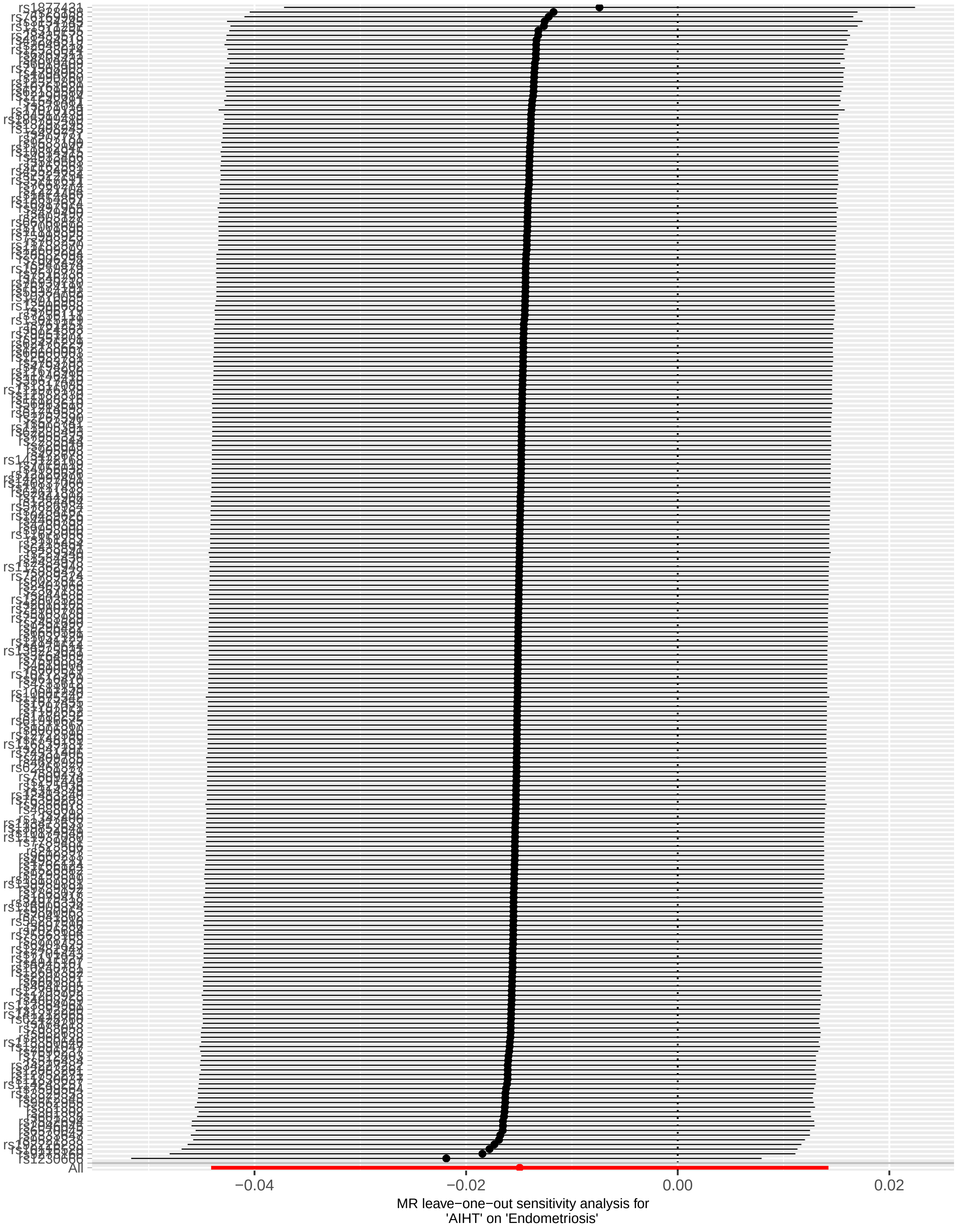
rs1877431
All
−0.04
−0.02
0.00
0.02
MR leave−one−out sensitivity analysis for
'AIHT' on 'Endometriosis'
