# Supplementary material for: Autoimmune hypothyroidism and endometriosis: evidence from bidirectional Mendelian randomization and an independent clinical cohort analysis
Source: Front Endocrinol (Lausanne). 2026 Aug 4;17:1898299. doi: 10.3389/fendo.2026.1898299 (PMC13487738; doi:10.3389/fendo.2026.1898299)
Supplement: Supplementary file 1 [file DataSheet1.zip › Supporting information/S6_Reverse leave-one-out plot.pdf]

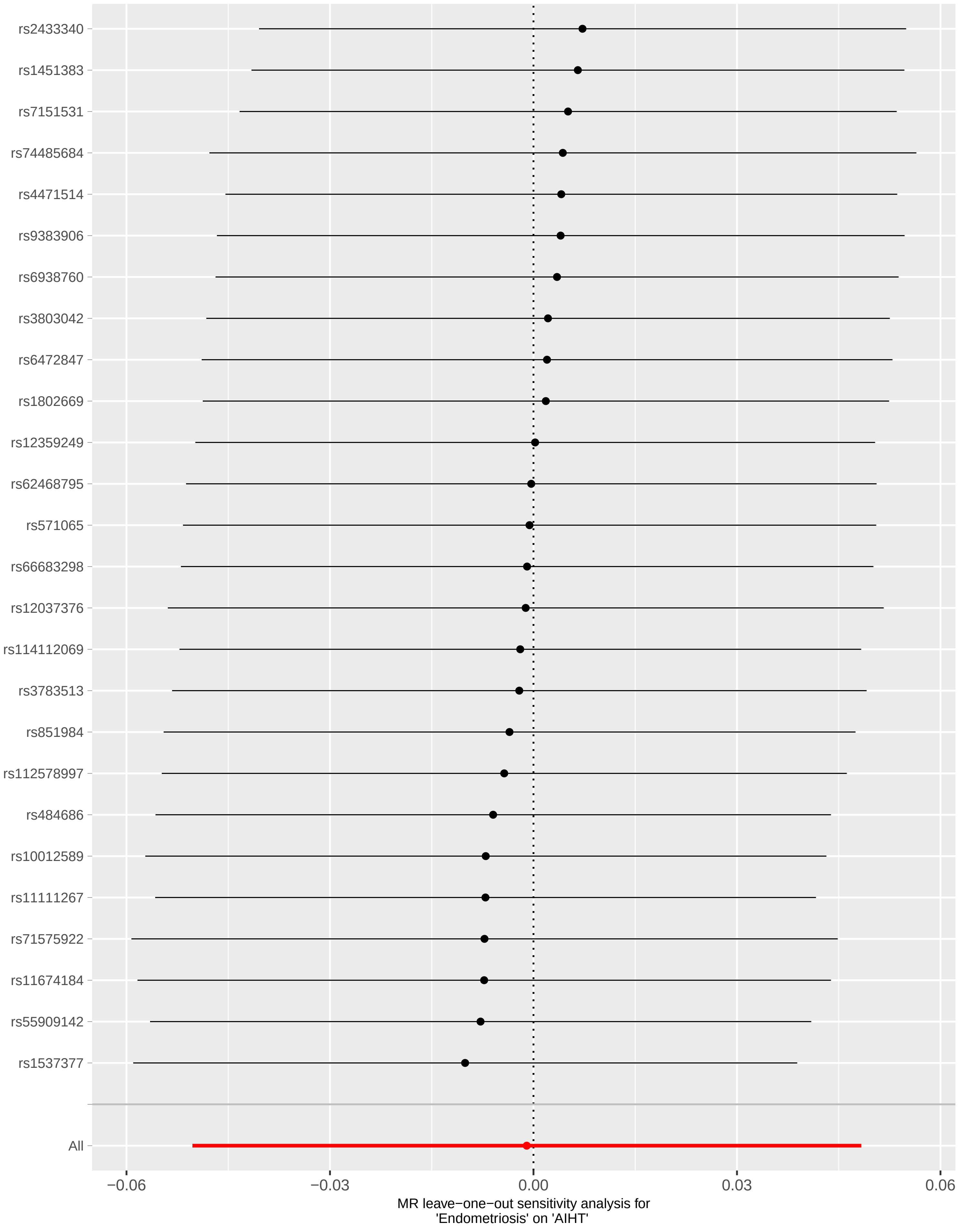

rs2433340
rs1451383
rs7151531
rs74485684
rs4471514
rs9383906
rs6938760
rs3803042
rs6472847
rs1802669
rs12359249
rs62468795
rs571065
rs66683298
rs12037376
rs114112069
rs3783513
rs851984
rs112578997
rs484686
rs10012589
rs11111267
rs71575922
rs11674184
rs55909142
rs1537377
All
−0.06
−0.03
0.00
0.03
0.06
MR leave−one−out sensitivity analysis for
'Endometriosis' on 'AIHT'
