# Supplementary material for: Autoimmune hypothyroidism and endometriosis: evidence from bidirectional Mendelian randomization and an independent clinical cohort analysis
Source: Front Endocrinol (Lausanne). 2026 Aug 4;17:1898299. doi: 10.3389/fendo.2026.1898299 (PMC13487738; doi:10.3389/fendo.2026.1898299)
Supplement: Supplementary file 1 [file DataSheet1.zip › Supporting information/S7_Forward single-SNP forest plot.pdf]

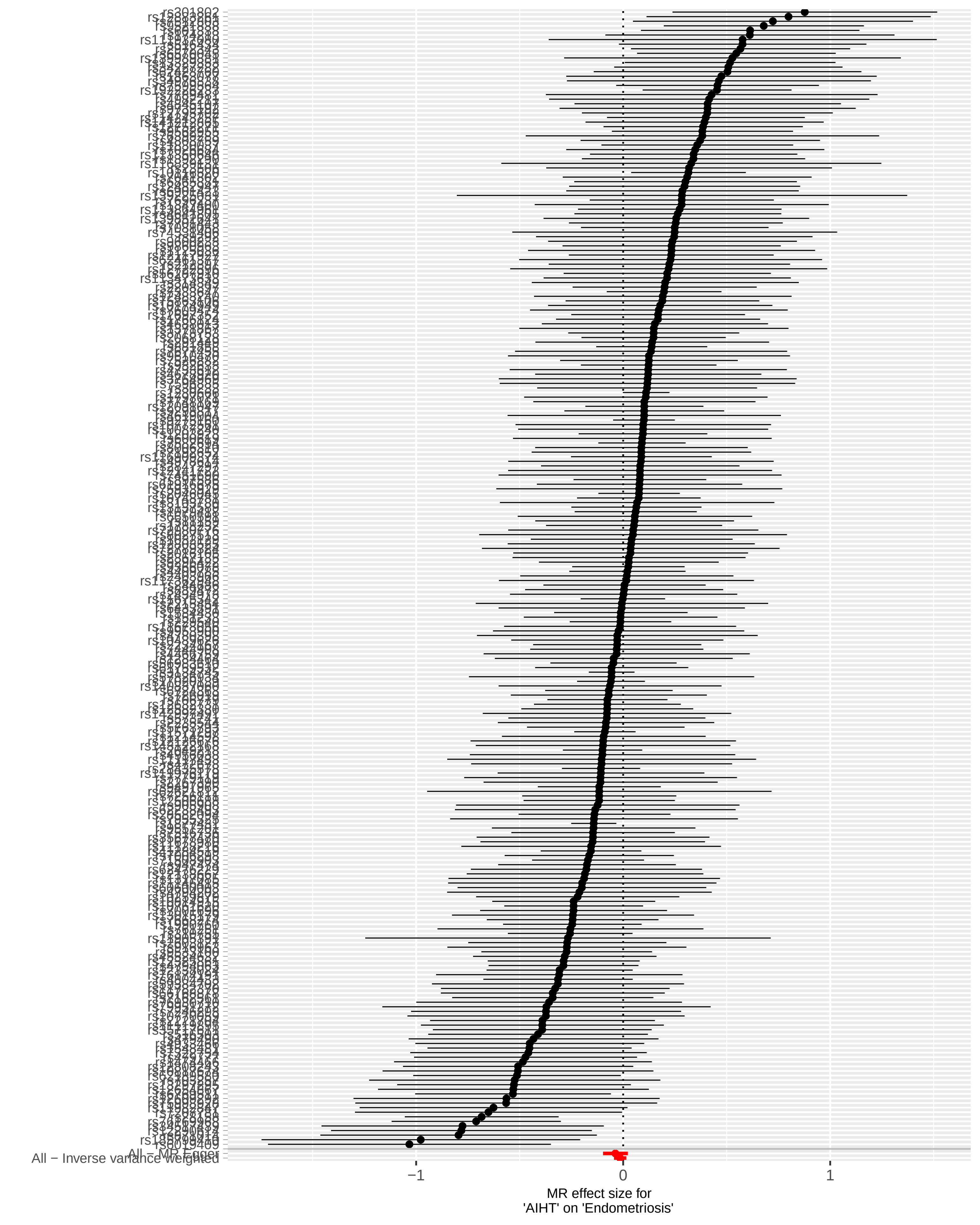

rs301802
All − MR Egger
All − Inverse variance weighted
−1
0
1
MR effect size for
'AIHT' on 'Endometriosis'
