# Supplementary material for: Autoimmune hypothyroidism and endometriosis: evidence from bidirectional Mendelian randomization and an independent clinical cohort analysis
Source: Front Endocrinol (Lausanne). 2026 Aug 4;17:1898299. doi: 10.3389/fendo.2026.1898299 (PMC13487738; doi:10.3389/fendo.2026.1898299)
Supplement: Supplementary file 1 [file DataSheet1.zip › Supporting information/S8_Reverse single-SNP forest plot.pdf]

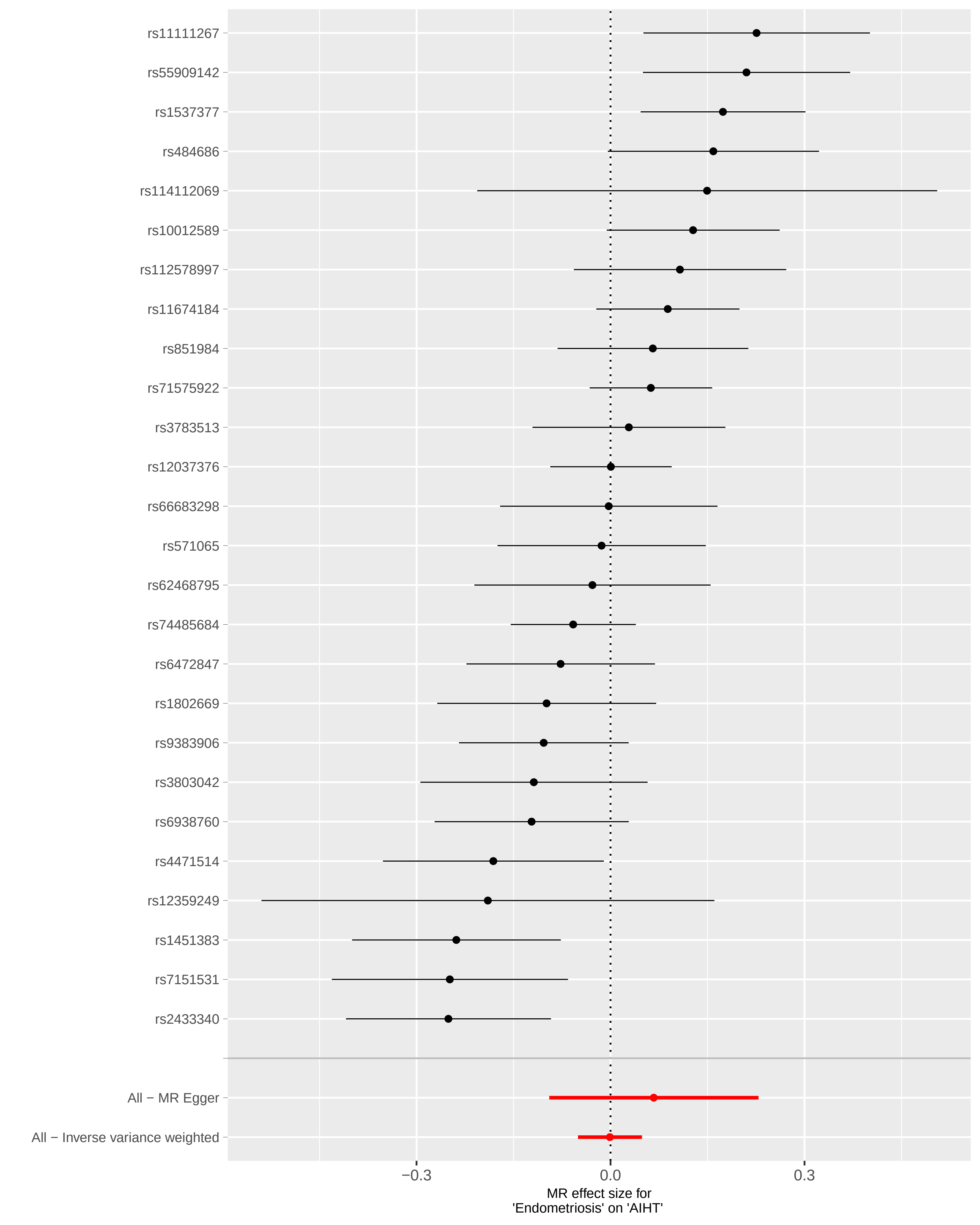
rs11111267
rs55909142
rs1537377
rs484686
rs114112069
rs10012589
rs112578997
rs11674184
rs851984
rs71575922
rs3783513
rs12037376
rs66683298
rs571065
rs62468795
rs74485684
rs6472847
rs1802669
rs9383906
rs3803042
rs6938760
rs4471514
rs12359249
rs1451383
rs7151531
rs2433340
All − MR Egger
All − Inverse variance weighted
−0.3
0.0
0.3
MR effect size for
'Endometriosis' on 'AIHT'
