# Supplementary material for: Autoimmune hypothyroidism and endometriosis: evidence from bidirectional Mendelian randomization and an independent clinical cohort analysis
Source: Front Endocrinol (Lausanne). 2026 Aug 4;17:1898299. doi: 10.3389/fendo.2026.1898299 (PMC13487738; doi:10.3389/fendo.2026.1898299)
Supplement: Supplementary file 1 [file DataSheet1.zip › Supporting information/Supporting information captions.docx]

## Supporting information captions

**Supplementary Table S1 | Harmonized instrumental variables included in the forward Mendelian randomization analysis.**
This table lists the harmonized genetic instruments retained for the forward autoimmune hypothyroidism-to-endometriosis Mendelian randomization analysis, together with SNP–exposure and SNP–outcome association estimates, allele information, SNP-specific F statistics, and approximate SNP-level variance explained (R²).

**Supplementary Table S2 | Harmonized instrumental variables included in the reverse Mendelian randomization analysis.**
This table lists the harmonized genetic instruments retained for the reverse endometriosis-to-autoimmune hypothyroidism Mendelian randomization analysis, together with SNP–exposure and SNP–outcome association estimates, allele information, SNP-specific F statistics, and approximate SNP-level variance explained (R²).

**Supplementary Table S3 | Genome-build liftOver audit. Part A summarizes mapping success and failure in both analysis directions; Part B lists variants that failed liftOver; Part C presents exploratory analyses of the regional clustering of mapping failures; and Part D provides methodological and interpretative notes. Because the audit used the raw pre-clumping candidate pool, nearby variants may be correlated, and the regional analyses should not be interpreted as formal evidence of biological enrichment.**

**Supplementary Table S4 | Statistical power calculations for the bidirectional Mendelian randomization analyses.**
This table presents the approximate statistical power of the forward and reverse Mendelian randomization analyses across assumed causal odds ratios, based on outcome sample size, case proportion, total variance explained by the genetic instruments, a two-sided alpha level of 0.05, and a binary-outcome Mendelian randomization power framework.

**Supplementary Table S5 | Complete bidirectional Mendelian randomization results including mode-based estimates.**
This table presents the complete causal estimates for the forward and reverse Mendelian randomization analyses using MR-Egger regression, weighted median, inverse-variance weighted, simple mode, and weighted mode methods.

**Supplementary Table S6 | MR-PRESSO sensitivity analyses of the bidirectional associations between autoimmune hypothyroidism and endometriosis.**
This table presents the MR-PRESSO global test results, identified outlier instruments, raw causal estimates, outlier-corrected estimates where applicable, and distortion-test results for the forward and reverse Mendelian randomization analyses.

**Supplementary Table S7 | Leave-one-out analysis results for the bidirectional Mendelian randomization analyses.**
This table presents leave-one-out analyses obtained by sequentially removing each instrumental variant and recalculating the inverse-variance weighted estimate. Part A presents the autoimmune hypothyroidism-to-endometriosis analysis, and Part B presents the endometriosis-to-autoimmune hypothyroidism analysis.

**Supplementary Table S8 | Phenome-wide association study lookup and manual review of instrumental variants.**
This table summarizes genome-wide significant phenome-wide associations identified for the harmonized instrumental variants, associated trait categories, minimum PheWAS P values, and final classifications after manual review as retained, flagged for sensitivity analysis, or excluded in sensitivity analysis.

**Supplementary Table S9 | PheWAS-informed sensitivity analyses excluding potentially pleiotropic variants.**
This table presents Mendelian randomization estimates and sensitivity diagnostics after PheWAS-informed exclusion of potentially pleiotropic variants. The supporting analyses include causal estimates, heterogeneity and MR-Egger intercept tests, exclusion summaries, lists of excluded variants, and methodological notes for the predefined filtering strategy.

**Supplementary Table S10 | HLA-region exclusion sensitivity analysis.**
This table presents sensitivity analyses after exclusion of variants located in the extended HLA region defined as chromosome 6:25,000,000–34,000,000 in GRCh37/hg19 coordinates. The supporting analyses include HLA-region variants, Mendelian randomization estimates, heterogeneity statistics, MR-Egger intercept results, and harmonized non-HLA instrument data.

**Supplementary Table S11 | Scenario-based assessment of the potential relative magnitude of participant-overlap-related weak-instrument bias.**
This table presents scenario-based estimates of the potential relative magnitude of weak-instrument bias under assumed participant-overlap proportions of 0%, 10%, 30%, 50%, and 100%. Estimates were calculated using the minimum observed SNP-specific F statistic in each Mendelian randomization direction as a conservative instrument-strength anchor.

**Supplementary Table S12 | Baseline characteristics of the independent clinical cohort.**
This table compares demographic, reproductive, gynecological, autoimmune, and thyroid-related baseline characteristics between women with confirmed endometriosis and diagnosis-negative controls in the independent infertility-clinic cohort.

**Supplementary Table S13 | Definitions and reference ranges for clinical thyroid outcomes.**
This table summarizes the institutional reference ranges, operational definitions, and analysis roles of thyroid-stimulating hormone, free thyroxine, thyroid peroxidase antibody, thyroglobulin antibody, thyroid autoimmunity, hypothyroidism, autoimmune hypothyroidism, subclinical hypothyroidism, overt hypothyroidism, and exclusionary thyroid conditions used in the clinical cohort analysis.

**Supplementary Table S14 | Sensitivity analyses in the independent clinical cohort.**
This table presents adjusted associations between endometriosis and thyroid-related outcomes in clinical sensitivity analyses excluding women with polycystic ovary syndrome, other autoimmune diseases, or adenomyosis, and in an analysis applying a stricter autoimmune hypothyroidism definition requiring thyroid autoimmunity together with elevated thyroid-stimulating hormone.

**Supplementary Figure S1. Scatter plot for the forward Mendelian randomization analysis.**
Scatter plot showing SNP-specific associations for genetic liability to autoimmune hypothyroidism and endometriosis risk. The fitted lines represent causal estimates derived from different Mendelian randomization methods.

**Supplementary Figure S2. Scatter plot for the reverse Mendelian randomization analysis.**
Scatter plot showing SNP-specific associations for genetic liability to endometriosis and autoimmune hypothyroidism risk. The fitted lines represent causal estimates derived from different Mendelian randomization methods.

**Supplementary Figure S3. Funnel plot for the forward Mendelian randomization analysis.**
Funnel plot assessing the distribution of SNP-specific estimates in the forward analysis of autoimmune hypothyroidism on endometriosis.

**Supplementary Figure S4. Funnel plot for the reverse Mendelian randomization analysis.**
Funnel plot assessing the distribution of SNP-specific estimates in the reverse analysis of endometriosis on autoimmune hypothyroidism.

**Supplementary Figure S5. Leave-one-out plot for the forward Mendelian randomization analysis.**
Leave-one-out analysis for the forward model evaluating the effect of genetic liability to autoimmune hypothyroidism on endometriosis risk. Each point represents the inverse-variance weighted estimate recalculated after excluding one SNP at a time.

**Supplementary Figure S6. Leave-one-out plot for the reverse Mendelian randomization analysis.**
Leave-one-out analysis for the reverse model evaluating the effect of genetic liability to endometriosis on autoimmune hypothyroidism risk. Each point represents the inverse-variance weighted estimate recalculated after excluding one SNP at a time.

**Supplementary Figure S7. Single-SNP forest plot for the forward Mendelian randomization analysis.**
Forest plot showing SNP-specific Wald ratio estimates for the forward analysis of autoimmune hypothyroidism on endometriosis.

**Supplementary Figure S8. Single-SNP forest plot for the reverse Mendelian randomization analysis.**
Forest plot showing SNP-specific Wald ratio estimates for the reverse analysis of endometriosis on autoimmune hypothyroidism.

**Supplementary File 1. R scripts used for the Mendelian randomization and clinical cohort analyses.**
This file contains the R scripts used for data processing, genome-build liftover, instrument selection, harmonization, Mendelian randomization analyses, sensitivity analyses, figure generation, and clinical cohort analyses.
